# A Model to Identify Candidates for Lymph Node Dissection Among Patients With High-Risk Endometrial Endometrioid Carcinoma According to Mayo Criteria

**DOI:** 10.3389/fonc.2022.895834

**Published:** 2022-06-20

**Authors:** Wen Lu, Xiaoyue Chen, Jingyi Ni, Zhen Li, Tao Su, Shuangdi Li, Xiaoping Wan

**Affiliations:** ^1^ Department of Gynecology, Shanghai First Maternity and Infant Hospital, School of Medicine, Tong Ji University, Shanghai, China; ^2^ Department of Clinical Research Center, Shanghai First Maternity and Infant Hospital, School of Medicine, Tong Ji University, Shanghai, China; ^3^ Department of Gynecology, The International Peace Maternity & Child Health Hospital of China Welfare Institute, Shanghai Jiaotong University, Shanghai, China

**Keywords:** endometrial, endometrioid carcinoma, lymph node dissection, Mayo criterion, molecular pathological markers, serum CA125

## Abstract

**Background:**

The Mayo criteria are the most widely accepted algorithm for predicting the risk of lymph node metastasis in endometrial endometrioid carcinoma (EEC). However, the clinical value of these criteria in high-risk patients is limited and inconclusive.

**Methods:**

A total of 240 patients with EEC meeting the Mayo high-risk criteria between January 1, 2015, and December 31, 2018 were included in our study. We retrospectively collected the laboratory reports, basic clinical information, clinicopathological and immunohistochemistry (IHC) findings, and the sequences of molecular pathological markers of these patients. A nomogram for predicting the likelihood of positive lymph node status was established based on these parameters.

**Results:**

Among the 240 patients, 17 were diagnosed with lymph node metastasis. The univariable analyses identified myometrial invasion >50%, aberrant p53 expression, microsatellite instable (MSI), and cancer antigen 125 (CA125) ≥35 U/ml as potential risk factors for lymph node metastasis. The multivariable analyses showed that aberrant p53 expression, MSI, and CA125 ≥35 U/ml were independent predictors of lymph node metastasis. The area under the curve (AUC) for the nomogram was 0.870, as compared to 0.665 for the Mayo criteria.

**Conclusions:**

Our novel prediction model effectively identifies patients at high risk for lymphatic metastasis. This model is a promising strategy for personalized surgery in patients with high risk according to the Mayo criteria.

## Introduction

Endometrial endometrioid carcinoma (EEC) is a surgically staged disease (1). Regional lymph node metastasis is the most important factor for determining prognosis and recommending treatment. Traditionally, primary surgical treatment includes total hysterectomy, bilateral salpingo-oophorectomy (TH/BSO), and standard lymph-node dissection (LND) (2). EEC is typically hormone sensitive and is often accompanied by moderate malignancy, along with obvious symptoms exhibited in early-stage disease. Thus, most patients are diagnosed at early stages without lymph node metastasis; the potential morbidity of routine LND may outweigh clinical benefits. Nowadays, whether and to what extent LND is necessary remain controversial. The acceptance and indications of LND vary among countries and facilities (3, 4).

The Mayo criteria are the most widely accepted algorithm for predicting the risk of lymph node metastasis in EEC (5, 6). They are largely based on specific preoperative and intraoperative clinicopathological findings (7) and categorize low-risk patients with EEC as those meeting the following characteristics: tumor diameter ≤2 cm, grade 1 or 2, and myometrial invasion (MI) ≤50%. In contrast, high-risk patients have tumors with >50% myometrial invasion, grade 3 histology, or tumor size >2 cm.

The reported lymph node involvement risk for patients classified as low and high risk according to the Mayo criteria were 1.4% and 6.4%, respectively (8). Most institutions in China today omit systematic LND in patients with EEC meeting the Mayo criteria for low risk. The Mayo criteria help avoid unnecessary systematic lymphadenectomy in patients with features of low-risk EEC. However, the clinical value of these criteria in high-risk patients is limited and inconclusive. Surgical staging with lymphadenectomy is routinely performed in most clinics in patients with high-risk EEC according to Mayo criteria; however, considerable overtreatment remains.

The molecular-based classification introduced by The Cancer Genome Atlas (TCGA) has initiated a new era and tremendous infusion of hope for individualized surgical treatment in endometrial cancer. A novel pragmatic molecular classifier using immunohistochemistry (IHC) on formalin-fixed, paraffin-embedded (FFPE) tissues has recently been validated (9). The Proactive Molecular Risk Classifier for Endometrial Cancer (ProMisE) identifies four molecular subtypes (10), which are analogous—but not identical—to the four genomic subtypes described in the Cancer Genome Atlas (11, 12). The ProMisE is reported to be a pragmatic molecular classifier to category endometrial cancers with different prognosis (10).

We retrospectively collected the laboratory reports, basic clinical information, clinicopathological and IHC findings, and the sequences of molecular pathological markers of patients with EEC meeting the Mayo high-risk criteria. We found that the combination of preoperative serum cancer antigen 125 (CA125) level and molecular parameters with Mayo criteria improved the prognostic accuracy of lymph node metastasis risk in patients with high-risk EEC per Mayo criteria. This investigation aimed to develop a modified model based on the Mayo criteria, with adequate accuracy to predict negative nodes and could be used to provide precise guidance on the scope of surgery in patients with high-risk EEC per Mayo criteria. To our knowledge, no similar research has yet been published.

## Material and Methods

### Study Population and Surgical Procedure

Data of patients with EEC who underwent surgical treatments at the Department of Gynecology at Shanghai First Maternity and Infant Hospital between January 2015 and December 2018 were retrospectively evaluated. The inclusion criteria were (1) EEC diagnosed by two gynecological pathologists, (2) complete clinical and pathological data, and (3) high risk according to the Mayo criteria. The exclusion criteria were (1) lymphadenectomy not performed during the primary surgery, (2) multiple primary tumors, and (3) patients administered neoadjuvant chemotherapy. Overall, 240 patients met the inclusion criteria. Written informed consent for the use of their biospecimens for research purposes was obtained from all patients before treatment. Research ethics approval for the tissue/biospecimen analysis and this project was granted by the review board of Shanghai First Maternity and Infant Hospital of Tongji University School of Medicine.

All patients underwent preoperative magnetic resonance imaging (MRI) to evaluate cervical invasion. Patients indicative of having gross cervical involvement received radical TH/BSO. Patients diagnosed with grade 3 disease underwent simultaneous paraaortic lymphadenectomy according to National Comprehensive Cancer Network (NCCN) guidelines. All operations were performed by the same gynecologic oncologist. The extent of the LND was the same regardless of the surgical technique (open or laparoscopic). Systematic pelvic lymphadenectomy included resection of the internal and external iliac, medial and lateral deep inguinal, obturator, sacral, and common iliac nodes. Para-aortic lymphadenectomy included the systematic resection of all nodes from the precaval, laterocaval, interaortocaval, preaortic, and lateroaortic areas up to the inferior mesenteric vein. Each specimen was collected separately according to its anatomical location for selective histopathological examination.

### Variables and Definitions

The patients’ tumors were staged according to the 2009 International Federation of Gynecology and Obstetrics staging system. Histological type was determined according to the World Health Organization classification.

The uterus was bisected to inspect the endometrial surface during frozen section. The tumor diameter was defined as the largest dimension of the lesion. In cases with more than one lesion, only the lesion with the largest diameter was considered. The extent of MI was categorized as ≤50% or >50%. For the frozen examination of MI, the uterus is bisected along the longitudinal axis and then serially sectioned from lower uterine segment to the fundus. Gross assessment is performed to figure out the lesion and to identify the area concerning for deepest invasion. Then, cancer tissues were biopsied carefully to ensure all tumor sites were included. Full-thickness representative sections are submitted for frozen section examination to assess the maximum depth of myometrial invasion.

Pretreatment serum CA125 level was determined by radioimmunoassay (RIA) (Abbott Diagnostics, Abbott Park, IL). The concentration was considered increased for values ≥35 U/ml.

The formalin-fixed and paraffin-embedded tissues from the hysterectomy specimens were collected for IHC analyses. Immunostaining was performed at the Pathology Department of Shanghai First Maternity and Infant Hospital. The immunostaining results were assessed independently by two pathologists blinded to the patient characteristics and outcome. Tissue sections were incubated overnight with primary antibodies against p53 (clone DO-7, 1:2,000, Neomarkers), MLH1 (clone ES05, 1:100, DAKO), MSH2 (clone FE11,1:100, DAKO), and MSH6 (clone EPR3945, 1:800, GENE TEX) at room temperature or with primary antibody PMS2 (clone EP51, 1:50, DAKO), anti-SPOP (ab81163, Abcam), ER (clone SP1, Denmark), and PR (clone IE2, Denmark) at 4°C. A linker (mouse linker, SM804, DAKO; rabbitlinker, SM805, DAKO) was used afterwards. A 30-min incubation with a secondary antibody (Poly-HRP-GAM/R/R; DPV0110HRP; ImmunoLogic) was then performed. DAB+ (K3468, DAKO) was used as chromogen, and sections were counterstained with hematoxylin. Immunostaining for p53 was considered aberrant if a completely negative or strongly positive staining was observed in >75% of tumor cells (nuclear or cytoplasmic). Mismatch repair protein status was also investigated. Tumors were considered microsatellite instable (MSI) if the tumor cells showed a loss of nuclear staining of at least one mismatch repair protein among MLH1, MSH2, MSH6, and PMS. Tumor cells exhibiting nuclear positivity for all mismatch repair proteins were categorized as mismatch-repair (MSS) positive. Estrogen receptor (ER) and progesterone receptor (PR) were scored as positive when at least 10% of tumor cells showed nuclear expression. For the identification of DNA polymerase epsilon, catalytic subunit (POLE) exonuclease domain hotspot mutations, Sanger sequencing was used to analyze exons 9, 12, 13, and 14 (13).

### Statistical Analysis

Descriptive statistics of the demographic and clinical–pathological characteristics are reported as frequencies and proportions for categorical variables and medians and interquartile ranges for continuous variables. Univariable logistic regression analyses were conducted to assess the potential predictors for lymph node metastasis. Next, all parameters significantly associated with lymph node metastasis in univariate analyses and variables that might be related to lymph node metastasis according to clinical relevance were included in the full multivariable model and were selected to develop the final nomogram.

The nomogram performance was assessed by discrimination and calibration. Discrimination in the current context was the ability to differentiate between women with and without lymph node metastasis. This assessment was performed using the receiver operating characteristic (ROC)-derived area under the curve (AUC). A calibration plot with 2,000 bootstrap replications was used to assess the agreement between the observed incidence and the nomogram-predicted probability. The optimal cutoff point of the nomogram was estimated by Youden’s J index. A decision-curve analysis (DCA) was used to determine the clinical net benefit associated with the use of the model.

All statistical tests were performed using IBM SPSS Statistics for Windows, version 22.0, and R statistical package v.3.4.4 (R Project for Statistical Computing, www.r-project.org). All tests were two-sided, with a significance level set at *p* < 0.05.

## Results

### Clinical Patient Characteristics

We identified 467 women who were eligible for the study between January 1, 2015, and December 31, 2018. Among these, 240 patients met the inclusion criteria. The demographic and clinical data of these 240 patients are presented in **Table 1**. The median age of the cohort was 55 years [interquartile range (IQR), 49.00–60.25 years]. Most patients were overweight, with a median body mass index (BMI) of 24.60 kg/m^2^ (IQR, 22.70–26.60 kg/m^2^). In this population, 223 patients (92.9%) were staged as between IA and IIIB, whereas 17 patients (7.1%) were diagnosed with advanced disease (IIIC–IV). All patients underwent pelvic lymphadenectomy, among whom 25 patients with G3 differentiation also underwent para-aortic lymphadenectomy. Among all patients, 17 (7.08%) were diagnosed with lymph node metastasis. Age at diagnosis, BMI, histology differentiation, and tumor diameter did not differ between patients with EEC with and without lymph node metastasis. MI, kg/m^2^, and cervix involvement differed between the two groups (all p<0.03).

**Table 1:** The demographics and pathological characteristics of the patients.

n	Overall	Negative Lymph Nodes	Positive Lymph Nodes	p
	240	223	17	
**Age**	55.00 [49.00, 60.25]	55.00 [50.00, 61.00]	50.00 [44.00, 58.00]	0.141
<60	167 (69.6)	154 (69.1)	13 (76.5)	0.714
≥60	73 (30.4)	69 (30.9)	4 (23.5)	
**BMI (median [IQR])**	24.60 [22.70, 26.60]	24.60 [22.70, 26.60]	25.50 [23.60, 26.60]	0.313
**FIGO 2009 stage**				
IA	152 (63.3)	152 (68.2)	0 (0.0)	<0.001
IB	42 (17.5)	42 (18.8)	0 (0.0)	
II	27 (11.2)	27 (12.1)	0 (0.0)	
IIIA	1 (0.4)	1 (0.4)	0 (0.0)	
IIIB	1 (0.4)	1 (0.4)	0 (0.0)	
IIIC1	15 (6.2)	0 (0.0)	15 (88.2)	
IIIC2	2 (0.8)	0 (0.0)	2 (11.8)	
**Histology**				
1–2	215 (89.6)	201 (90.1)	14 (82.4)	0.548
3	25 (10.4)	22 (9.9)	3 (17.6)	
**Primary tumor size**				
<20 mm	9 (3.8)	9 (4.0)	0 (0.0)	0.856
≥20 mm	231 (96.2)	214 (96.0)	17 (100.0)	
**Myometrial invasion**				
≤50%	173 (72.1)	166 (74.4)	7 (41.2)	0.008
>50%	67 (27.9)	57 (25.6)	10 (58.8)	
LVSI				
Negative	197 (82.1)	196 (87.9)	1 (5.9)	<0.001
Positive	43 (17.9)	27 (12.1)	16 (94.1)	
**Involving cervix**				
Negative	206 (85.8)	195 (87.4)	11 (64.7)	0.026
Positive	34 (14.2)	28 (12.6)	6 (35.3)	
**p53**				
Normal	224 (93.3)	213 (95.5)	11 (64.7)	<0.001
Aberrant	16 (6.7)	10 (4.5)	6 (35.3)	
**dMMR**				
MSS	187 (77.9)	179 (80.3)	8 (47.1)	0.004
MSI	53 (22.1)	44 (19.7)	9 (52.9)	
**Ca125**				
<35	182 (75.8)	176 (78.9)	6 (35.3)	<0.001
≥35	58 (24.2)	47 (21.1)	11 (64.7)	
**POLE**				
No mutation	212 (88.3)	197 (88.3)	15 (88.2)	1
Mutation	28 (11.7)	26 (11.7)	2 (11.8)	
**PR**				
<10%	34 (14.2)	32 (14.3)	2 (11.8)	1
≥10%	206 (85.8)	191 (85.7)	15 (88.2)	

N, number; BMI, body mass index; IQR, interquartile range; LVSI, lymph vascular space invasion.

### Univariable and Multivariable Models Predicting Lymph Node Metastasis

The univariable analyses identified MI >50% [odds ratio (OR), 4.160; 95% confidence interval (CI), 1.527–11.947), aberrant p53 expression (OR, 11.618; 95%CI, 3.442–37.778), MSI (OR, 4.577; 95%CI, 1.660–12.853), and CA125 ≥35 (OR, 6.865; 95%CI, 2.481–20.840) as potential risk factors for lymph node metastasis (**Table 2**, all p<0.01). All these variables, and histological grade (for clinical relevance consideration), were included in the multivariable logistic regression model. The multivariable analyses showed that aberrant p53 expression (OR, 12.661; 95%CI, 3.006–57.364), MSI (OR, 4.414; 95%CI, 1.331–15.326), and CA125 ≥35 (OR, 5.309; 95%CI, 1.563–20.013) were independent predictors of lymph node metastasis. The nomogram also included histological grade and MI because of their clinical relevance. The results of the univariate and multivariable analyses are presented in **Table 2**.

**Table 2:** Univariate and multivariate analysis of lymph node metastasis.

	Univariable analyses		Multivariable analyses	
	OR (95% CI)	P value	OR (95% CI)	p-value
**Histological grade**				
1–2	1.0		1.0	
3	1.958 (0.428, 6.590)	0.319	1.700 (0.320, 7.149)	0.491
**Myometrial invasion**				
<50%	1.0		1.0	
≥50%	4.160 (1.527, 11.947)	0.006^*^	2.067 (0.609, 7.051)	0.238
**p53**				
Normal	1.0		1.0	
Aberrant	11.618 (3.442, 37.778)	<0.001^*^	12.661 (3.006, 57.364)	0.001^*^
**dMMR**				
MSS	1.0		1.0	
MSI	4.577 (1.660, 12.853)	0.003^*^	4.414 (1.331, 15.326)	0.015^*^
**CA125**				
<35	1.0		1.0	
≥35	6.865 (2.481, 20.840)	<0.001^*^	5.309 (1.563, 20.013)	0.009^*^
**POLE**				
No mutation	1.0			
Mutation	1.010 (0.154, 3.860)	0.99		
**PR**				
<10%	1.0			
≥10%	1.257 (0.333, 8.212)	0.769		
**LVSI**				
Negative	1.0			
Positive	116.148 (22.359, 2,138.550)	<0.001		

*P < 0.05.

### Development and Validation of the Prediction Model

**Figure 1:**
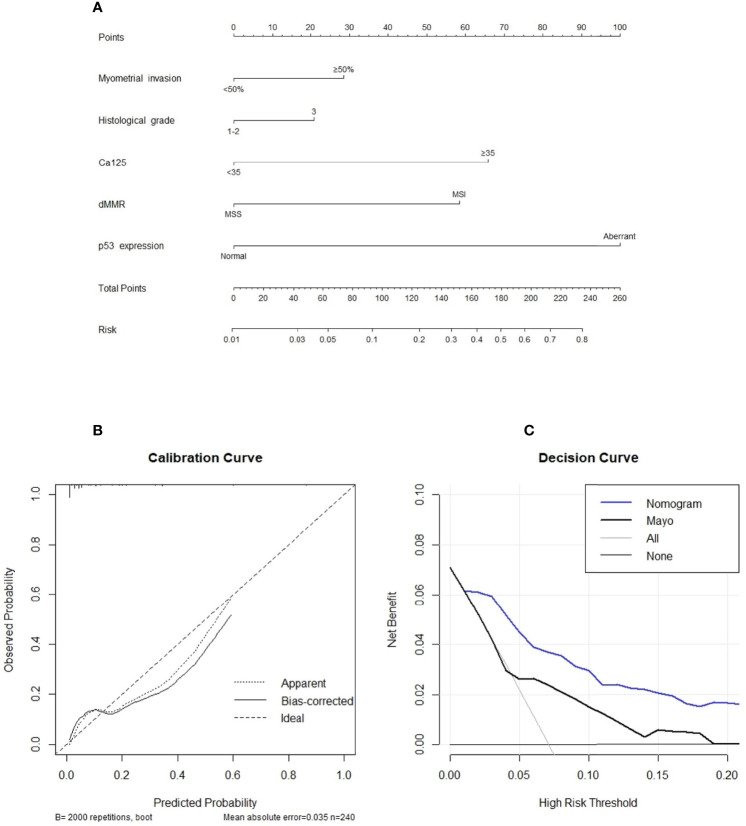
**(A)** A nomogram for predicting the likelihood of positive lymph node status. To use the nomogram, the value for each predictor is determined by first drawing a line upward to the point reference line. The points are then summed and a line is drawn downward from the total points line to determine the predicted probability of node positivity. **(B)** Calibration plot of the observed proportions and predicted probabilities of lymph node metastasis based on the novel nomogram. The predicted probability of pathological lymph node invasion aligns closely with the actual probability. **(C)**, decision curve analyses demonstrating the net benefit associated with the use of the novel nomogram for the detection of lymph node metastasis.

**Figure 2:**
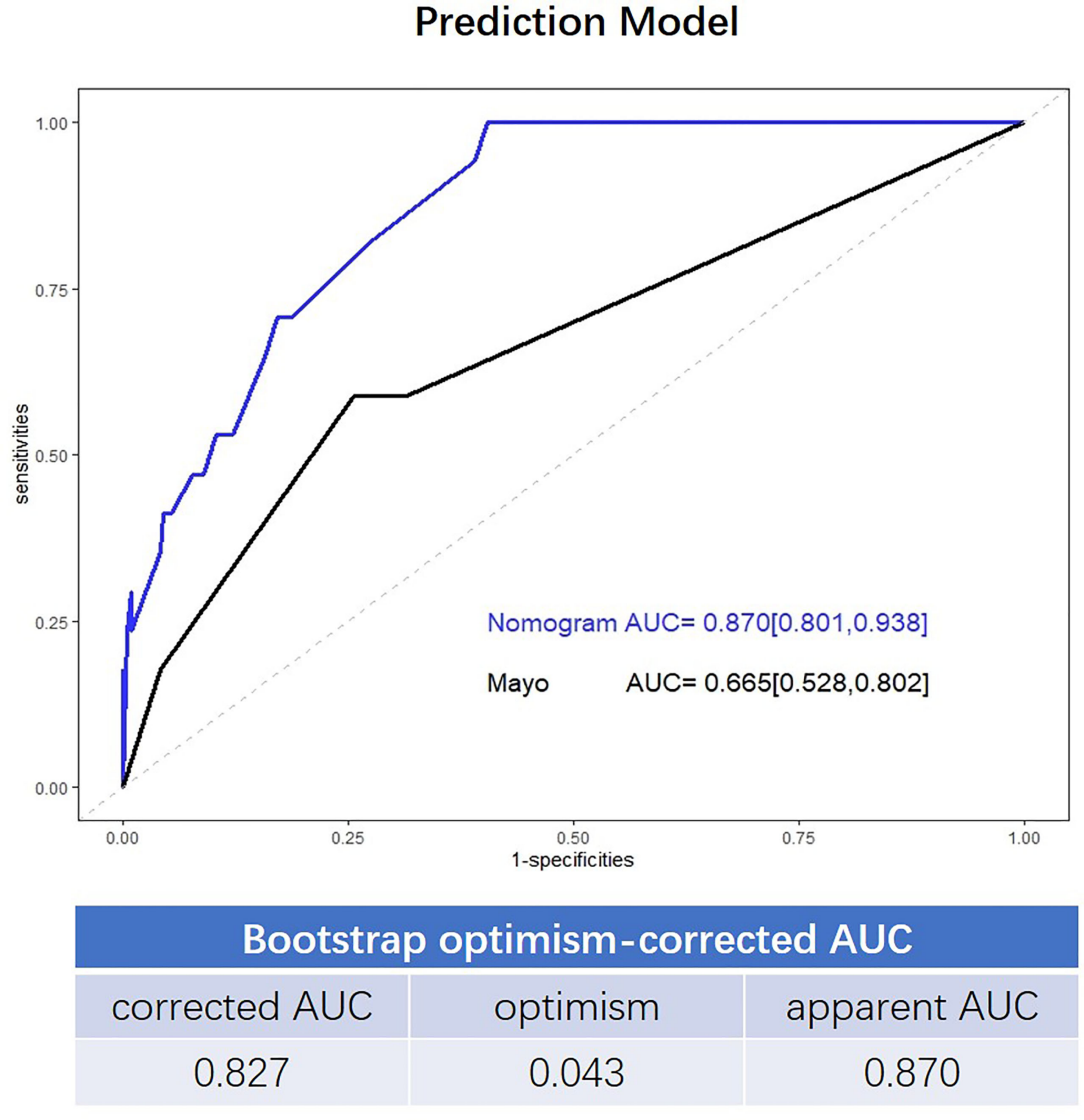
Receiver operating characteristic (ROC) curve showing the performance of the Mayo criteria and novel model.

The final nomogram is as shown in **Figure 1A** and depicts the multivariable effect of each variable on lymph node metastasis. The calibration plot of the predicted probabilities against the observed probabilities of lymph node metastasis indicated excellent concordance (**Figure 1B**). The DCA demonstrated that our nomogram improved clinical risk prediction against the Mayo criteria by comparing the net benefit to a threshold probability of 0–20% (**Figure 1C**). The AUC for the nomogram was 0.870 (95% CI, 0.801–0.938), whereas the bootstrap optimism-corrected AUC was 0.827 as compared to 0.665 (95% CI, 0.528–0.802) for the Mayo criteria (**Figure 2**).

**Table 3** lists the errors associated with the use of the novel model to predict lymph node metastasis. Using 3% as the best cutoff point (43 points in the nomogram), 133 unnecessary lymphadenectomies would have been spared, and all patients with lymph node metastasis were taken into account.

**Table 3:** Systematic analyses of the nomogram-derived cutoffs used to discriminate between patients with or without histologically confirmed lymph node metastasis.

Probability of LNM, cutoff(%)	Patients above cutoff with histologically approved LNM	Patients below cutoff without histologically approved LNM	Patients above cutoff without histologically approved LNM	Patients below cutoff with histologically approved LNM	Sensitivity	Specificity	Positive predicted value	Negative predicted value
1	17	0	223	0	1	0	0.07083	
2	17	106	117	0	1	0.475336	0.12687	1
3	17	133	90	0	1	0.596413	0.15888	1
4	16	136	87	1	0.941176	0.609865	0.15534	0.9927
5	14	161	62	3	0.823529	0.721973	0.18421	0.98171
6	12	181	42	5	0.705882	0.811659	0.22222	0.97312
7	12	181	42	5	0.705882	0.811659	0.22222	0.97312
8	12	183	40	5	0.705882	0.820628	0.23077	0.9734
9	11	188	35	6	0.647059	0.843049	0.23913	0.96907
10	11	188	35	6	0.647059	0.843049	0.23913	0.96907

### Histopathological and Molecular Concordance of Endometrial Tissues From Resected Uterus or Curettage Samples

Histology and p53, and deficient mismatch repair (dMMR) staining were analyzed in endometrial tissues from either the bisected uterus or preoperative curettage specimens. We observed a high consistency between these samples, as shown in **Table 4**. The concordance rates for histology, p53 expression, and dMMR were 94.3%, 92.9%, and 84.5%, respectively.

**Table 4:** Concordance of histopathological features and molecular alterations in endometrial hysterectomy and pre-operative specimens.

	Hysterectomy *n* = 142 (%)	Curettage *n* = 142 (%)	Total discordant cases	Concordance rate
**Histology**				
Endometrioid grade 1–2	134 (94.3)	132 (92.9)	8	94.3
Endometrioid grade 3	8 (5.7)	10 (7.1)		
**p53**				
Normal	125(88.1)	126 (88.7)	10	92.9
Aberrant	17(11.9)	16 (11.3)		
**dMMR**				
MSS	107 (75.6)	107 (75.6)	22	84.5
MSI	35 (24.4)	35 (24.4)		

## Discussion

The diagnostic accuracy of lymph node status in endometrial cancer is an important issue. Although surgical staging is the golden standard, LND is controversial in EEC because of its long-term morbidity, uncertain treatment value, and high negative lymph node metastasis rate in histology. The accuracy of the existing lymph node metastasis risk models is not satisfactory. Even according to the most accepted Mayo risk-adopted algorithm, more than 70% of patients without lymph node metastasis were overtreated with unnecessary LND. This investigation is the only study of patients with high-risk EEC according to Mayo criteria to structure a model for the assessment of the risk of lymph node metastasis. We developed a novel model by retrospectively analyzing the relationship of lymph node metastasis with preoperative CA125 levels, traditional histology findings, and molecular indicators. Our study further divided high-risk patients per Mayo criteria into two subgroups: those less likely to experience lymph node metastasis and those more likely to have positive lymph nodes in lymphadenectomy. Our novel model helped 55.42% of patients with high-risk EEC according to Mayo criteria avoid LND, and all patients with lymph node metastasis were taken into account.

The Mayo criteria comprise three indicators: tumor size, MI depth, and differentiation. However, in the current study, tumor size was not associated with the lymph node status. This may be partly attributed to the fact that the sizes of most cancers in our study were ≥20 mm (231/240, 96.2%). Tumor size in our study was defined as the diameter of the largest dimension of the lesion in the bisected uterus, which was more accurate than radiological modalities. The signal of the inner zone of endometrial carcinoma is often higher than that at the margin; thus, evaluating tumor size on MRI might ignore the lesion periphery, leading to a smaller measured tumor size than the actual size (14). Similarly, the MI depth was estimated intraoperatively, which was reportedly significantly better than MRI in determining deep MI (15–17). Although the tumor grades did not differ between the lymph nodes metastasis or non-metastasis groups, it was included in our prediction model for clinical relevance consideration.

In contrast to the Mayo criteria, our prediction model included dMMR and p53 expression. The factors were surrogate markers of microsatellite instability and low copy number subgroups of endometrial cancer, as defined by the TCGA, which were associated with intermediate and unfavorable prognoses, respectively (18). The results of our study showed that dMMR and p53 expression were associated with lymph node metastasis in both univariable and multivariable analysis, a finding consistent with those reported previously (19–21). Moreover, elevated serum CA125 level was also associated with lymphatic metastasis in EEC (22, 23), which was also verified by our study. Incorporation of these parameters into the prediction model could improve its performance for the discrimination of low-risk patients among those with Mayo high-risk factors. ROC curve analysis showed that the prediction accuracy of our novel algorithm was 0.870 (95%CI, 0.801–0.938), which was superior to that of the Mayo criteria (AUC=0.665, 95% CI, 0.528–0.802).

Oncologists have attempted to tailor lymphadenectomy according to the combinations of multiple clinical indicators in EEC. For instance, American researchers have established a risk-scoring system for the individualized prediction of lymphatic dissemination. A set of pathological variables, namely, MI, grade, primary tumor diameter, cervical stromal invasion, and lymph-vascular space invasion (metastasis) were incorporated into the nomogram. The internal validation of the nomogram showed good discrimination (AUC=0.88) (24). French oncologists have further provided external validation of this nomogram (25). The predictive accuracy according to the discrimination of the AUC criteria was 0.64 for the nomogram. Recently, several studies have managed to combine clinicopathological parameters and molecular indicators to predict lymph node metastasis in EEC (20, 26–29). It was showed that incorporating molecular indicators can predict lymph node metastasis more accurately (20). However, POLE and MMR are important parameters in the molecular-based classification introduced by TCGA. No published nomogram included these molecular markers. This study addressed this gap based on the integration of traditional pathological parameters with genomic findings to aid doctors in determining treatment.

Our model did not include lymph-vascular space invasion (LVSI) for several reasons. First, to be utilized as a prediction model, LVSI should be diagnosed by frozen section. However, time constraints, limited sampling, and technical artifacts might lead to erroneous interpretation. A relatively low agreement (68.3%) has been observed for the comparison of LVSI diagnosed by frozen section with that diagnosed by permanent section (30). Second, although LVSI has gained a prominent position in most risk stratification systems for EC (31, 32), the reproducibility among pathologists in the presence (or absence) of LVSI is the Achilles heel of histology diagnosis, with poor reported reproducibility of LVSI assessment and grading in EEC (33). The high variability of LVSI suggests that it cannot be used as a reliable component of the prediction model. Finally, based on the current model, we hope to screen for suitable factors in curettage samples to establish a feasible prediction model through the current model. It is impossible to obtain LVSI information from curettage specimens.

Sentinel lymph node (SLN) mapping is another proposed research path to identify patients at risk for lymph node metastasis (34, 35). However, the requirement for special dyes and imaging systems has impeded its widespread implementation (36). Moreover, mapping failure is not rare and can be caused by lymphatic obstruction, obesity, surgeon expertise, or the depth of cervical injection (37). Most importantly, the accuracy of this technique remains controversial since its first mention in the NCCN in 2014 (38). In our previous study, the overall sensitivity of the SLN to identify nodal metastatic disease was 50% (95% CI, 17.4–82.5), whereas the negative predictive value (NPV) and false negative (FN) rate were 96.6% (95%CI 91.0–98.9) and 50%, respectively. We concluded that SLN mapping was not sensitive and had a high FN rate for node metastasis in endometrial cancer with high-risk histology (39). Our novel model is superior to SLN in both cost reduction and accessibility. Most importantly, no patients below the cutoff had been histologically confirmed for lymph node metastasis according to our novel model.

This study has several limitations. First, this was a single-center retrospective study; thus, the model requires validation in other cohorts in different centers. Second, only 17 lymph node metastases occurred in our cohort. In addition, external validation was not performed. This was due to the reason that lymph node metastasis rate is low in EEC and the relatively small sample size of our trial. Further assessment in prospective studies were needed. Third, the detection of MSI and p53 expression was performed in postoperative resection specimens rather than curettage specimens. The ideal prediction model would be based on the genomic findings of curettage specimens to accurately discriminate patients at high risk for lymph node metastasis. However, we instead compared the molecular alterations in endometrial specimens obtained from the resected uterus or curettage and observed a high concordance between these specimens (93.5% for p53, 84.5% for dMMR). Our finding highlighted the potential use of curettage specimens to predict lymphatic metastasis.

## Conclusion

In conclusion, our novel prediction model effectively identified patients at high risk for lymphatic metastasis. This model is a promising strategy for personalized surgery in patients with high risk according to the Mayo criteria. Further studies are needed to assess the feasibility of this prediction model in preoperative curettage specimens.

## Data Availability Statement

The original contributions presented in the study are included in the article/**Supplementary Material**. Further inquiries can be directed to the corresponding authors.

## Ethics Statement

The studies involving human participants were reviewed and approved by the ethics committee of Shanghai First Maternity and Infant Hospital (Approval No. 2017026). The patients/participants provided their written informed consent to participate in this study.

## Author Contributions

XW has full access to all data and takes responsibility for the study accuracy and originality. Study concept and design: WL and TS. Manuscript drafting: XC and SL. Statistical analysis: JN and ZL. Critical revision of the manuscript for important intellectual content: XW and SL. All authors contributed to the article and approved the submitted version.

## Funding

This research was granted by the Chinese National Natural Science Foundation (81971338, 81972438, and 82172975) and by the Clinical Science and Technology Innovation Project of Shanghai Shenkang Hospital Development Center (SHDC12020107).

## Conflict of Interest

The authors declare that the research was conducted in the absence of any commercial or financial relationships that could be construed as a potential conflict of interest.

## Publisher’s Note

All claims expressed in this article are solely those of the authors and do not necessarily represent those of their affiliated organizations, or those of the publisher, the editors and the reviewers. Any product that may be evaluated in this article, or claim that may be made by its manufacturer, is not guaranteed or endorsed by the publisher.
