# Supplementary material for: A Model to Identify Candidates for Lymph Node Dissection Among Patients With High-Risk Endometrial Endometrioid Carcinoma According to Mayo Criteria
Source: Front Oncol. 2022 Jun 20;12:895834. doi: 10.3389/fonc.2022.895834 (PMC9251056; doi:10.3389/fonc.2022.895834)
Supplement: Supplementary file 1 [file Table_1.docx]

Supplementary Table 1.Primer of the POLE

| POLE-Exon9 | Forward | 5'-GTGTTCAGGGAGGCCTAATG-3' |
| --- | --- | --- |
|  | Reverse | 5'-CCATCCCAGGAGCTTACTTC-3' |
| POLE-Exon13 | Forward | 5'-CCTGGCTTCTGTTCTCATTCT-3' |
|  | Reverse | 5'-GATGTGGCTCACATGCCT-3' |
| POLE-Exon14 | Forward | 5'-GACCCTGGGCTCTTGATTT-3' |
|  | Reverse | 5'-GGACATCCACCTCCATTCAG-3' |
